# Dose Dependent Dual Effect of Baicalin and Herb Huang Qin Extract on Angiogenesis

**DOI:** 10.1371/journal.pone.0167125

**Published:** 2016-11-30

**Authors:** Dongqing Zhu, Shanshan Wang, John Lawless, Jianchen He, Zhengui Zheng

**Affiliations:** 1 Department of Physiology, School of Medicine, Southern Illinois University Carbondale, Carbondale, Illinois, United States of America; 2 Department of Pharmacy, Jiangsu Food & Pharmaceutical Science College, Huai’an, Jiangsu Province, People’s Republic of China; 3 School of Basic Medical Sciences, Shanghai University of Traditional Chinese Medicine, Shanghai, People’s Republic of China; University of Michigan, UNITED STATES

## Abstract

Huang Qin (root of *Scutellaria baicalensis*) is a widely used herb in different countries for adjuvant therapy of inflammation, diabetes, hypertension, different kinds of cancer and virus related diseases. Baicalin is the main flavonoid in this herb and has been extensively studied for 30 years. The angiogenic effect of herb Huang Qin extract and baicalin was found 13 years ago, however, the results were controversial with pro-angiogenic effect in some studies and anti-angiogenic effect in others. In this paper, the angiogenic effect of baicalin, its aglycone form baicalein and aqueous extract of Huang Qin was studied in chick embryo chorioallantoic membrane (CAM) model. Dose dependent dual effect was found in both aqueous extract and baicalin, but not in baicalein, in which only inhibitory effect was observed. In order to reveal the cellular and molecular mechanism of how baicalin and baicalein affect angiogenesis, cell proliferation and programmed cell death assays were performed in treated CAM. In addition, quantitative PCR array including 84 angiogenesis related genes was used to detect high and low dosage of baicalin and baicalein responsive genes. Low dose baicalin increased cell proliferation in developing blood vessels through upregulation of multiple angiogenic genes expression, but high dose baicalin induced cell death, performing inhibitory effect on angiogenesis. Both high and low dose of baicalein down regulated the expression of multiple angiogenic genes, decreased cell proliferation, and leads to inhibitory effects on angiogenesis.

## Introduction

Herb Huang Qin (root of *Scutellaria baicalensis*) is commonly used in traditional medicine in China and some other countries. It is officially listed in Chinese Pharmacopoeia with broad effects such as purging fire, cleaning away heat, moistening aridity, detoxifying toxicosis, stoppage of bleeding and preventing miscarriage [1]. In addition, Huang Qin was also used in more than 30 different formulas [1]. Pharmacological reports in recent years have indicated that *S*. *baicalensis* has a multitude of medicinal properties, including anti-inflammatory, antidiabetic, antiviral, antihypertension, antioxidant, and anticancer effects [2–4]. Close to 300 compounds were isolated from this herb, including flavonoids, alkaloids, phytosterols and some others, among these chemicals, flavonoids with the form of glycosides were the most abundant [5, 6]. Many flavonoids from *S*. *baicalensis* are pharmacologically active and show great potential in the treatment of inflammation and different kinds of cancer [7, 8]. Baicalin (5,6,7-trihydroxyflavone) is one of the main active ingredients and accounts for more than 5% total dry mass in *S*. *baicalensis* [9, 10]. In the current Chinese Pharmacopeia, baicalin is selected as the quality control marker of herb Huang Qin [1].

Angiogenesis, the process of forming new blood vessels, is a fundamental step in development and physiology, such as wound healing, organ growth and reproduction, as well as key step in pathological conditions like chronic inflammation, tumor progression and metastasis [11–13]. It is a major component of cancer and heart diseases [14]. Among many angiogenesis assay methods, the CAM assay is commonly used in experiments involving tumor angiogenesis and validation the potential function of modulators of angiogenesis [15, 16]. Angiogenesis inhibitors benefit cancer control, while angiogenesis promoters are useful in some ischemic disease therapy [17].

It is generally accepted today that tumor growth is an angiogenesis-dependent process, which requires an increase of vascular growth. Tumors lacking angiogenesis remain dormant, and rapid growth of tumors follows the formation of new blood vessels and acquisition of blood supply [18, 19]. Dr. Folkman in the early 1970s first proposed using angiogenesis inhibitors as anticancer drugs [20], and in 2004 the US Food and Drug Administration (FDA) approved an angiogenesis inhibitor Bevacizumab as second-line treatment of colorectal cancer [21]. Since then there is a great interest in identifying and modulating anti-angiogenic pathways and development of anti-angiogenic drug for therapeutic purposes. Angiogenesis could be inhibited directly by targeting endothelial cells in the growing vasculature or indirectly by targeting tumor cells themselves or stromal cells associated to tumor. So Angiogenesis inhibitors can be classified as direct endogenous inhibitors of angiogenesis, such as angiostatin, tumstatin and many others, and indirect inhibitors of angiogenesis, which could block the activity of pro-angiogenic proteins, such as Iressa and Bevacizumab, including conventional chemotherapeutic agents like flavonoids from medicinal plants [22].

On the other hand, induction of therapeutic angiogenesis has been developed to treat ischemic diseases like cardiovascular disease [23]. Therapeutic angiogenesis aims to induce angiogenic response in order to re-vascularize ischemic tissues using growth factors such as VEGF, FGF, IGF-1 and others, as well as agencies, which can increase growth factors expression [24]. In recent years, stem cell based therapy and stem cell combined gene therapy have been used in ischemic animal models [25]. Several therapeutic strategies have been proposed and tested even at the clinical level [26]. A potential method may be the use of drugs with angiogenic activity, available in an oral formulation, which are currently being administered to patients for treatment of different ischemic conditions [27].

Many natural products extracted from plants show potential pro-angiogenesis or anti-angiogenesis effect [28–30]. Publications in the past 15 years suggest that *S*. *baicalensis* extract has strong inhibitory effect on disease related angiogenesis in different models [7, 28, 31–33]. Extracts of *S*. *baicalensis* strongly inhibits cell growth and proliferation in different cancer cells [34, 35], and anticancer function of baicalein has been found both *in vitro* and *in vivo* [36, 37]. Extracts of *S*. *baicalensis* also has the potential to treat diseases and conditions that require angiogenesis, including wound healing, tissue repair for cardiovascular and other ischemic diseases [38–40]. The angiogenic effect of baicalin in published literature is controversial. In 2003, Liu et al and several others reported that baicalin and its aglycone form, baicalein, were potential inhibitors of angiogenesis, but the pro-angiogenesis effect of baicalin has also been reported [40–44].

There are only a few publications reporting the mechanism of how angiogenic process was affected by baicalin or baicalein. Jo et al found that high-dose baicalin showed a significant reduction in the expression of matrix metalloproteinase-2 (MMP-2), MMP-9, angiotensin II, and vascular endothelial growth factor (VEGF) [42], Liu et al also revealed that baicalein and baicalin treatment resulted in a dose-dependent decrease of MMP-2 activity, cell proliferation and apoptotic changes in cultured human umbilical vein endothelial cells [41]. Some studies on anticancer activities demonstrated that baicalin, baicalein or Huang Qin extract suppresses the angiogenesis of tumor cells through Wnt/β-catenin, TGF-β, PI3K/Akt pathways or NF-κB signaling [7, 45, 46]. On the other hand, baicalin and Huang Qin extract were also found to induce VEGF expression through the activation of the ERRα pathway [40]. As Huang Qin and its main components, baicalin and baicalein are commonly used in herbal medicine, it’s important to confirm their effect and discover the mechanism on angiogenesis.

In this study, dose-dependent dual effect of baicalin and Huang Qin aqueous extract on angiogenesis was revealed. High dosage of baicalin or baicalein showed anti-angiogenesis effect through induction of apoptosis, but low dosage of baicalin was found to promote angiogenesis through increasing cell proliferation. Possible genetic mechanism underlying the dual effect in different dosages of baicalin was studied using pathway specific PCR array.

## Materials and Methods

### Herb and extract preparation

The herb Huang Qin was purchased from Chinese herbs direct (http://www.chineseherbsdirect.com) and identified by Prof. Jianchen He at Shanghai University of Traditional Chinese Medicine, it is the root of *Scutellaria baicalensis*. Aqueous extract was prepared by soaking the herb in distilled water at room temperature for 1 hour, and then boiling for another hour. The solution was centrifuged (12 000 g, 10 min) to remove any insoluble parts, the supernatant was carefully measured and 1gram dry weight per milliliter stock solution was prepared and stored in -20°C freezer until use. Just before the CAM experiments, the extract was diluted into different concentrations using distilled water. Baicalin hydrate (Cat. No. 375756) and baicalein (Cat. No. 465119) were purchased from Sigma-Aldrich. The stock solutions were prepared with DMSO, and working solutions were made by dilution with distilled water before each use. Final concentration of DMSO in working solution was 5%, so a 5% DMSO water solution was used as control.

### Chick chorioallantoic membrane (CAM) assay

The fertilized chicken eggs were purchased from University of Illinois at Urbana–Champaign chicken farm and incubated at 37°C in a automatic rocking egg incubator with the humidity at 40–60%. On the 3rd day of incubation, the eggs were windowed with forceps, then sealed with a tape and put back into the incubator with windowed side up. The incubator was not allowed to rock after that. Additional water was also added in the water chamber of the incubator to ensure the proper humidity (40–60%) was maintained. On day 7.5 of incubation, a sterilized filter paper containing 10μl of Huang Qin aqueous extract, baicalin or baicalein solution was applied to the CAM surface. The eggs were then returned to the incubator. 48 hours later, an appropriate volume of a fresh made fixative (methanol: acetone = 1:1 (v/v)) was injected using a 28-gauge needle into the 9.5-day-old CAM and fixed for 30min at room temperature. The CAM was cut out from eggs and photographs were taken using a Leica stereomicroscope. The numbers of vessels were observed and small vessels (≤0.2mm) radially converging toward the center were counted. Sample size: baicalein group n = 30, all other groups: n = 40.

### Morphometric analysis

10 samples for each group were selected from collected CAMs, and were fixed overnight in 4% formaldehyde (PFA) solution at 4°C, followed by dehydration (in alcohol serials), clearing and infiltration stages, then embedded in paraffin. 6μm sections were cut and slides with CAM tissue were prepared. Thickness of the CAM was measured from haematoxylin and eosin stained sections with a calibrated objective at 40× magnification using 10×10 calibrated grids in the10× ocular. Samples close to the carrier region (within 5mm to the edge of carrier) were selected and each selected CAM sample was measured at 4 different locations and the results were averaged [47].

### HPLC analysis

The HPLC system consisted of a solvent delivery pump (Waters 600, USA), an automatic injector (Waters 2767, USA), and a 2489 UV/Visible (UV/Vis) Detector (Waters 2489, USA). A Kinetex C18 column (5μm, 150 x 4.6 mm, Phenomenex) was used. The HPLC condition was modified from Gao et al [48]. In brief, elution of the samples and standards were performed using 0.1% formic acid (eluent A) and methanol (eluent B). The gradient elution initial conditions were 50% of eluent B with linear gradient to 60% from 2 to 10 min, followed by linear gradient to 80% of eluent B at 30 min, and then linear gradient to 99% of eluent B at 31 min, this proportion being maintained for 2 min. The column was then returned to the initial condition at 33 min and maintained until the end of the run at 35 min. The flow rate was 1 ml/min. The injection volume was 5μl and three injections were performed for each standard and 5 injections for Huang Qin aqueous extract. The quantitation of baicalin and baicalein was based on standard curves of the published method [48, 49].

### Cell death, and cell proliferation assays

For cell death assay, LysoTracker Red DND-99 (Life Technologies) was used to label the apoptotic cells and the method was modified from previously described protocols [50, 51]. CAM was cut and removed from eggs before fixation. After being washed twice for 5min each in warm PBS (37°C), CAM was incubated in 5μM LysoTracker staining solution at 37°C for 30 min. After 3–4 times warm PBS wash, The CAM was fixed in 4% PFA overnight, then dehydrated through a methanol series (50%, 75%, 80%, 100%, 5–10 min each step) to eliminate background staining [50]. LysoTracker stained CAM samples can be stored indefinitely in methanol at -20°C, protected from light. Before imaging, the CAM samples were rehydrated through methanol series to PBS, and carefully mounted on slides, and a confocal microscope (Leica DM5500) was used for imaging. Sample size for each treatment group: n = 5.

For cell proliferation analysis, CAM was cut and removed from eggs before fixation, washed twice in PBS for 5 min each to remove blood, and then fixed in 4% PFA overnight at 4°C. A rabbit anti-phospho-histone H3 (PHH3) polyclonal antibody (Millipore Cat. #06–570) was used to label dividing cells [51]. We performed PHH3 whole mount immunofluorescence on CAM using a method modified from Ahnfelt-Ronne et al and Alanentalo et al [52, 53]. In short, after fixation, CAM was washed 3 times in PBS with 1% Triton, 30 min each time, then blocked (PBS+1% Triton + 10% goat serum) for at least 2 hours at room temperature. After washing away the blocking solution, the CAM was incubated for 3 days in PHH3 primary antibody (diluted 200 times) at 4°C, followed by 1 day wash in PBS +1% Triton solution, and then incubated in a secondary antibody for 2 days at 4°C. After being stained in DAPI solution for 1 day at 4°C, CAM was cleared in glycerol and mounted on slides, a confocal microscope (Leica DM5500) was used for imaging. Sample size for each treatment group: n = 5.

### Quantitative RT-PCR

Quantitative RT-PCR was modified from previously described methods [51, 54]. Total RNA was extracted from CAM with different treatments using TRIzol^®^ reagent (Invitrogen) following previous described protocol [55]. cDNA was made using iScript cDNA Synthesis Kit (Bio-rad) after quality control analysis of RNA using a Nanodrop spectrophotometer (ND2000, Thermo Scientific). Pathway-specific gene expression was then determined using the chicken angiogenesis PCR array (PAGG-024Z; Qiagen) and the CFX384 Touch Real-Time PCR System (Bio-rad) according to the manufacturer’s protocol. The complete list of genes assayed on the array can be found at the manufacturer's website (http://www.sabiosciences.com/rt_pcr_product/HTML/PAGG-024Z.html). Sample size for each treatment group: n = 4.

### Statistical analysis

As the comparison in this study was made between baicalin, baicalein or Huang Qin aqueous extract group to control group, all group differences in our dependent variables were revealed using Student's t-tests (one dependent variable between groups), significant level (P value) was set at 0.05.

## Results

### Baicalin and baicalein concentrations in aqueous extract of herb Huang Qin

**Fig 1:**
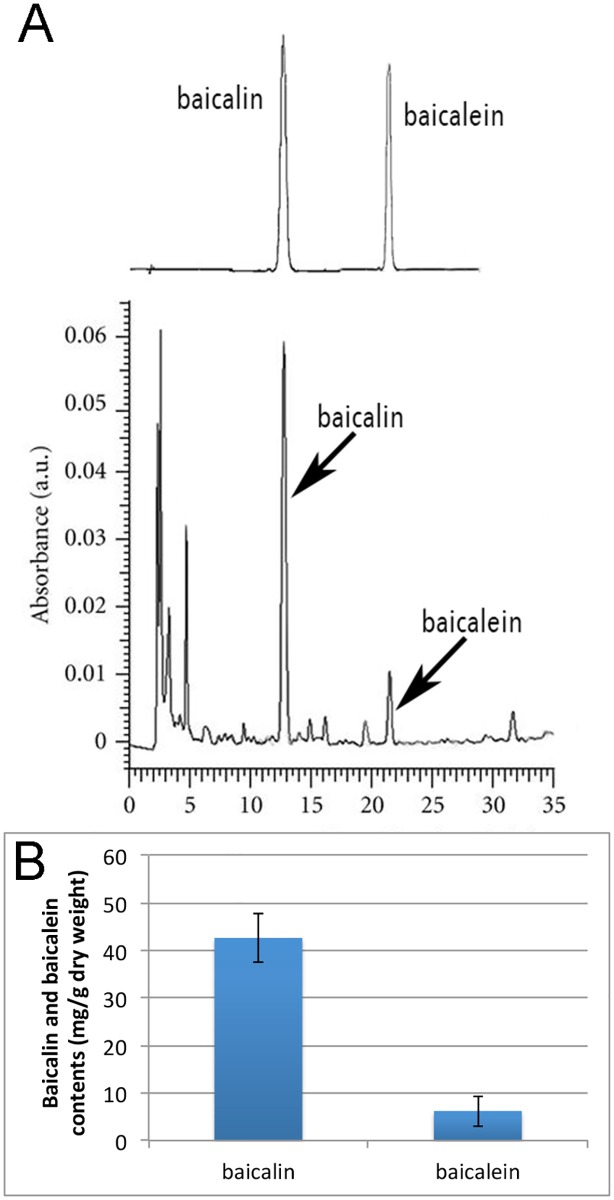
Determination of baicalin and baicalein contents in aqueous extract of herb Huang Qin using HPLC. A, HPLC profile of baicalin and baicalein in aqueous extract diluted with methanol before loading, the top is the standards. Retention time of baicalin and baicalein was 12.5 and 21.5 min respectively. B, baicalin and baicalein contents in herb Huang Qin aqueous extract. The data were the average of 5 repeats (n = 5), and the error bars show standard error (SE).

To evaluate the effect of herb extract of Huang Qin and its main active components, baicalin and baicalein contents in our aqueous extract of Huang Qin were quantified by HPLC. Fig 1 showed the results of HPLC analysis, with the retention time of baicalin and baicalein being 12.5min and 21.5 min in our system (Fig 1A). The average baicalin concentration in our aqueous extract was 42.6mg/g dry weight, while baicalein concentration was relatively low, only 7.2mg/g dry weight (Fig 1B).

### Dose dependent effect of Huang Qin aqueous extract, baicalin and baicalein on angiogenesis in CAM model

**Fig 2:**
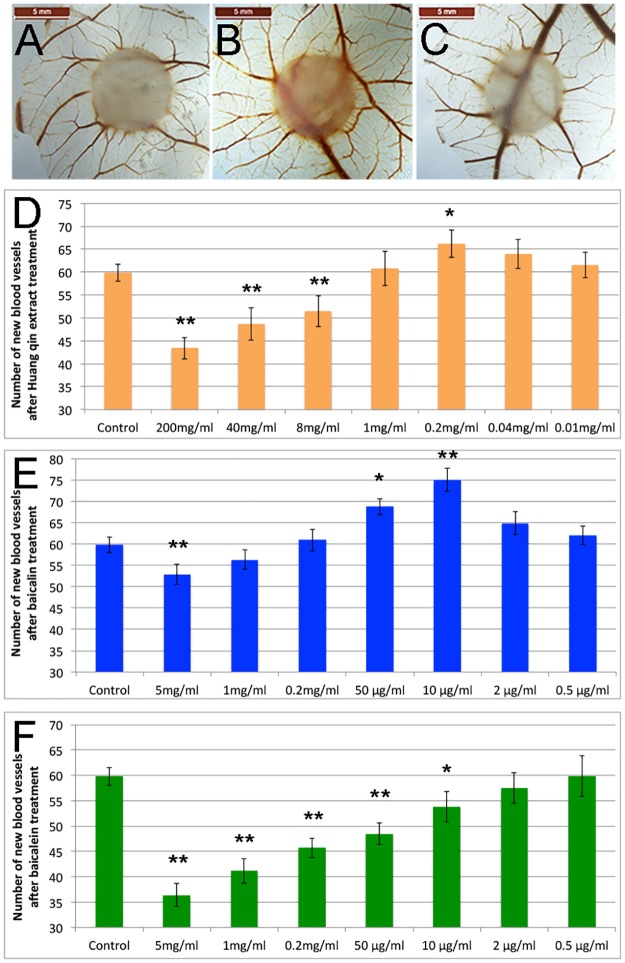
Dose dependent effect of Huang Qin extract, baicalin and baicalein on angiogenesis in CAM model. A-C, Selected CAM photographs of control (A), 10μg/ml baicalin (B) and 5mg/ml baicalin (C). D-F, Numbers of blood neo-vessels showing dose dependent angiogenic effects of Huang Qin aqueous extract (D), baicalin (E) and baicalein (F). 10μl of each solution was added on the carrier at embryonic day 7.5 of CAM, all small blood vessels (diameter ≤0.2mm) around the carrier were counted. Sample size: baicalein group n = 30, all other groups: n = 40. The scale bars in A, B and C are 5mm. The error bars in D, E and F show mean ±SE. The asterisks denote significant differences between each treatment group and control group (*p≤0.05, **P≤0.01).

Using CAM model, we first compared the newly formed blood vessel numbers between different dosages of Huang Qin aqueous extract groups. The results were shown in Fig 2D and S1 Fig. We could see that treatment of 200mg/ml, 40mg/ml and 8mg/ml extract in CAM caused significant reduction in blood vessels when compared to controls (p≤0.003), and 200mg/ml had the minimum blood vessels, only 43 neo-vessels on average, much less than control group, which were about 60. When the dosage of Huang Qin extract was reduced to 1 mg/ml, the number of blood vessels showed no significant difference between the treated and the control groups (p = 0.662). Interestingly, when CAM was treated with 0.2mg/ml Huang Qin aqueous extract, the newly formed blood vessels were significant increased (p = 0.017), it was still slightly more than control when the dose was reduced to 0.04mg/ml (but no significant difference, p = 0.073). When treated with even lower dose (0.01mg/ml), the average number of neo-blood vessels (62) was similar to control group (P = 0.305).

As the content of baicalin in our aqueous extract of Huang Qin was about 4% (Fig 1), we selected 5mg/ml baicalin as the highest dose to test its effect on angiogenesis in CAM model. The results revealed that 5mg/ml baicalin significantly inhibited blood vessel formation (P = 0.0001), but no significant effect was observed at doses of 1mg/ml (p = 0.165) and 0.2 mg/ml (p = 0.379) (Fig 2A–2C and 2E and S1 Fig). When we reduced the baicalin dosage to 50μg/ml and 10μg/ml, increased blood vessel numbers (68 and 74 respectively) were observed in treated CAM with either dosage (p≤0.004). No significant effect was found in 2μg/ml ((p = 0.079) or 0.5μg/ml (p = 0.125) baicalin treated group (Fig 2E and S1 Fig). The results in Fig 2E suggested that baicalin has a dual effect on angiogenesis; it inhibits angiogenesis at high concentrations (5mg/ml or above), but promotes angiogenesis at low concentrations (10–50μg/ml).

We also tested the angiogenic effect of baicalein in CAM model using the same dosages as baicalin, the results were presented in Fig 2F and S1 Fig. 5mg/ml baicalein showed a strong anti-angiogenic activity, and the quantities of newly formed blood vessels were decreased to 36, about a half of control group (p = 0.00038). From 5mg/ml to 50μg/ml dosages, baicalein treated CAM groups showed significantly fewer blood vessels than those in the control group (p≤0.0004). The result suggested that baicalein showed a strong inhibitory effect on angiogenesis. Unlike baicalin, which had pro-angiogenic effect at low dosages (10–50μg/ml), baicalein significantly inhibited blood vessel formation (p = 0.025) as low as 10μg/ml dose (Fig 2F). When even lower dosages (2μg/ml and 0.5μg/ml) were tested, the inhibitory effect was not significant. In our experimental dosage range (0.5μg/ml - 5mg/ml), no pro-angiogenic effect of baicalein had been found. The results suggested that baicalein doesn’t show dual effect on angiogenesis.

During the study, we observed that treatment with different dosages of baicalin and baicalein changed CAM morphology. The CAMs after high dose of baicalin, baicalein or Huang Qin aqueous extract treatment were thinner, had fewer connective tissue cells and blood vessels (S2A–S2D and S2G Fig). On the contrary, treatment with low dose of baicalin induced more well-organized blood vessels and a large number of cells in both chorionic and allantoic layers, resulted in increased CAM thickness (S2A, S2E and S2G Fig).

### High dosage of baicalin and baicalein inhibit angiogenesis through induction of apoptosis

**Fig 3:**
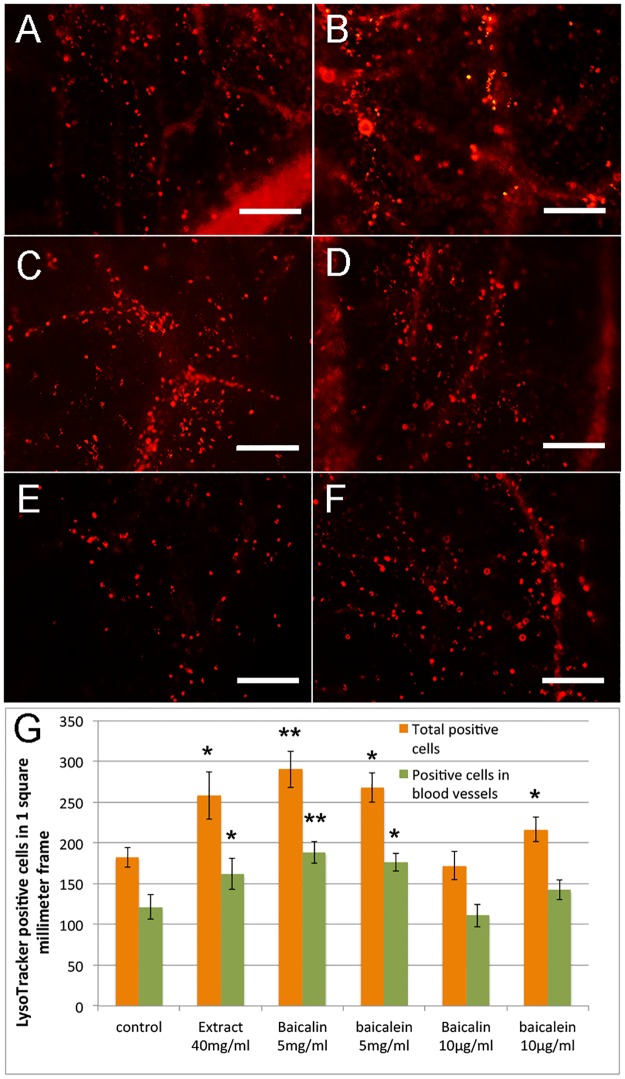
LysoTracker red staining showing programed cell death in Huang Qin extract, baicalin and baicalein treated CAM. A-F, Selected photographs showing LysoTracker positive cells (red) in control (A), 40mg/ml Huang Qin aqueous extract (B), 5mg/ml baicalin (C), 5mg/ml baicalein (D), 10μg/ml baicalin (E) and 10μg/ml baicalein (F) treated CAM. G, Comparison of LysoTracker positive cells in a 1 square millimeter frame of CAM in different treatments. The scale bars in A-F are 60μm. Graph in G showing average positive cells in a center layer of 1 square millimeter frame. 5 CAMs were selected from each treatment group, and in each CAM sample, the center layer of 4 different frames in 4 directions of CAM close to the carrier were counted. Error bars show mean ±SE, and asterisks denote significant differences between each treatment group and control group (*P ≤ 0.05, **P ≤ 0.01).

After determining the effect on angiogenesis of baicalin, baicalein and aqueous extract of Huang Qin, we then tried to elucidate the mechanism underlying the anti-angiogenic effect of baicalein and high dose of baicalin in CAM. We performed apoptosis analysis using LysoTracker Red staining to detect programmed cell death in high (5mg/ml) and low (10μg/ml) doses of baicalin and baicalein treated CAM. Compared with controls, the number of total LysoTracker positive cells in CAM (P = 0.093) and positive cells in blood vessels only (P = 0.105) had no significant difference between low dose baicalin group and control (Fig 3A, 3E and 3G). Low dose baicalein treated CAM exhibited increased total apoptotic cells (P = 0.034), but the LysoTracker positive cells in blood vessels only (P = 0.058) had no significant difference (Fig 3A, 3F and 3G). High dose of baicalin (P = 0.004) and baicalein (p = 0.022) treatment significantly increased cell death in all cell types of CAM and in blood vessels only as well (Fig 3A, 3C, 3D and 3G). In addition, we found that 40mg/ml Huang Qin aqueous extract treatment also significantly (P = 0.016) increased cell death in blood vessels and other cells in CAM (Fig 3A, 3B and 3G). The results suggested that high dose of baicalin and baicalein inhibit angiogenesis through induction of apoptosis in CAM; low dose of baicalin and baicalein have no effect on apoptosis in blood vessel cells of CAM.

### Baicalein and low dose of baicalin affect angiogenesis through modulation of cell proliferation

**Fig 4:**
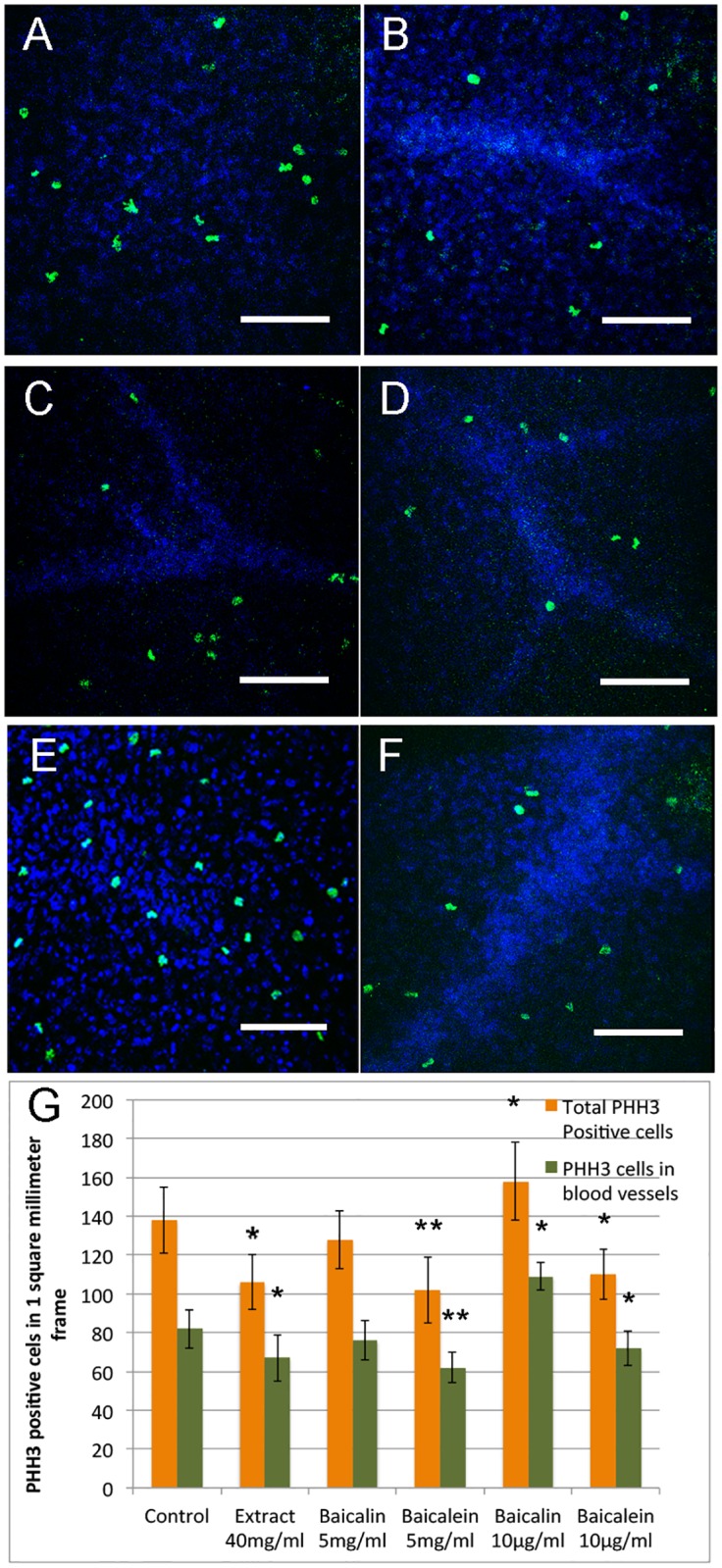
PHH3 immunofluorescence staining showing cell proliferation in Huang Qin extract, baicalin and baicalein treated CAM. A-F, Selected photographs of PHH3 positive cells in control (A), 40mg/ml Huang Qin aqueous extract (B), 5mg/ml baicalin (C), 5mg/ml baicalein (D), 10μg/ml baicalin (E) and 10μg/ml baicalein (F) treated CAM. PHH3: green, Dapi: blue. The scale bars in A-F are 30μm. Graph in G showing positive cells in a center layer of 1 square millimeter frame. 5 CAMs were selected from each treatment group, and in each CAM sample, the center layer of 4 different frames in 4 directions of CAM close to the carrier were counted. Error bars show mean ±SE, and asterisks denote significant differences between each treatment group and control group (*P ≤ 0.05, **P ≤ 0.01).

To understand how did baicalein and low dose of baicalin affect angiogenesis in CAM, we performed whole mount immunofluorescence assay using mitosis-specific antibody anti-phospho histone H3 (PHH3), and the results were shown in Fig 4. The amount of PHH3 positive cells in blood vessels of CAM were significantly reduced in high (p = 0.005) and low dose (p = 0.035) of baicalein, and 40mg/ml Huang Qin aqueous extract (p = 0.018) treated groups (Fig 4A, 4D, 4F and 4G). Interestingly, high dose baicalin treatment caused a slight reduction of PHH3 positive cells (no statistic difference, p = 0.054) compared to controls (Fig 4A, 4C and 4G), but in low dose baicalin treated group, the amount of PHH3 positive cells was significantly (p = 0.038) increased (Fig 4A, 4E and 4G). The results suggested that baicalein and low dose of baicalin affect angiogenesis by modulation of cell proliferation.

### Baicalin and baicalein regulate angiogenic gene expression in CAM

**Fig 5:**
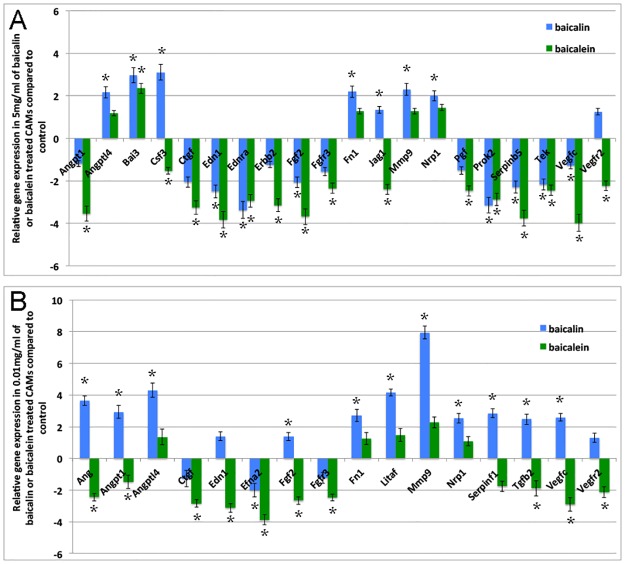
Relative expression of angiogenic genes in high and low dose of baicalin and baicalein treated CAM. (A) Relative transcript levels (*y*-axis shows fold changes) in high dose of baicalin and baicalein treated CAM compared to controls. Control is assigned a value of 0. Twenty-seven of 84 genes showed statistically significant changes of >2.0-fold (*P* < 0.05) to respond high dose of baicalin (12 genes) and baicalein (15 genes) treatment. (B) Relative transcript levels (fold changes) in low dose of baicalin and baicalein treated CAMs compared to controls. Twenty of 84 genes showed statistically significant differences of >2-fold (*P* < 0.05) to respond low dose of baicalin (11 genes) and baicalein (9 genes) treatment. Error bars show mean ±SE, and asterisks denote significant differences between each treatment group and control group (*P ≤ 0.05, **P ≤ 0.01). Sample size for each treatment: n = 4.

Based on the discovery that baicalin had a dose-dependent dual effect on angiogenesis, it induced apoptosis, inhibited cell proliferation at high doses, and promoted cell proliferation at low doses, we tested the hypothesis that the angiogenic gene network might be regulated differentially between high and low doses of baicalin. Given chicken angiogenesis PCR array has 84 angiogenic factors and other genes involved in angiogenesis including cytokines, growth factors and receptors, adhesion molecules, proteases inhibitors and other matrix genes, it enabled us to quickly and reliably analyze the expression of the focused panel of genes related to angiogenesis using real-time PCR. Quantification of the transcript levels of 84 genes in 48 hours high dose of baicalin and baicalein treated CAM identified 12 genes with significant (≥2 fold up or down regulation, P<0.05) responses to baicalin and 15 genes to baicalein (Fig 5A). High dose of baicalin treatment significantly down-regulated the transcripts levels of *EDN1*, *EDNRA*, *FGF2*, *PROK2*, *SERPINB5* and *TEK*, but up-regulated the expression of *ANGPTL4*, *BAI3*, *CST3*, *FN1*, *MMP9* AND *NRP1* (Fig 5A). On responding to high dose of baicalein, the expression level of *ANGPT1*, *CTGF*, *EDN1*, *EDNRA*, *ERBB2*, *FGF2*, *FGFR3*, *JAG1*, *PGF*, *PROK2*, *SERPINB5*, *TEK*, *VEGFC* and *VEGFR2* were down regulated compared to controls, while only *BAI3* was up regulated (Fig 5A). To recognize whether the same genes transcripts were regulated in CAM treated with low dose of baicalin and baicalein, we analyzed the relative expression levels of the same 84 genes in low dose of baicalin and baicalein treated CAM, and 11 genes with significant (≥2 fold up or down regulation, P<0.05) responses to baicalin and 9 to baicalein were revealed (Fig 5B). Among 11 genes responsive to low dose of baicalin, *ANG*, *ANGPT1*, *ANGPTL4*, *FN1*, *LITAF*, *MMP9*, *NRP1*, *SERPINF1*, *TGFB2 AND VEGFC* were up regulated, while only *EFNA2* was down regulated (Fig 5B). Low dose of baicalein treatment significantly down-regulated the transcript levels of *ANG*, *CTGF*, *EDN1*, *EFNA2*, *FGF2*, *FGFR3*, *VEGFC* and *VEGFR2*, only *MMP9* expression was significantly (≥2 fold, P<0.05) up regulated (Fig 5B).

## Discussion

**Fig 6:**
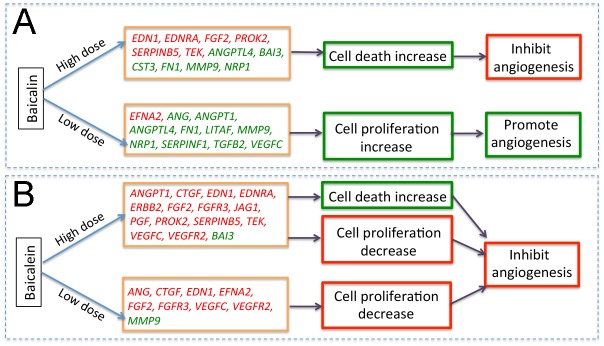
Potential mechanisms underlying dose dependent effect of baicalin and baicalein on angiogenesis. Schematic drawing shows potential mechanism of high and low dose of baicalin (A) and baicalein (B) affecting angiogenesis in CAM model. Green boxes indicate increased activity, and red boxes indicate decreased activity. In orange boxes, green texts show up regulated genes, and red texts show down regulated genes. High dose of baicalin down regulated *EDN1*, *EDNRA*, *FGF2*, *PROK2*, *SERPINB5*, *TEK*, and up regulated *ANGPTL4*, *BAI3*, *CST3*, *FN1*, *MMP9* and *NRP1*, increased cell death, and performed inhibitory effect on angiogenesis (A). Low dose of baicalin down regulated *EFNA2*, but up regulated *ANG*, *ANGPT1*, *ANGPTL4*, *FN1*, *LITAF*, *MMP9*, *NRP1*, *SERPINF1*, *TGFB2* and *VEGFC*, increased cell proliferation and resulted an angiogenesis promoting effect (A). High dose of baicalein down regulated the expression of *ANGPT1*, *CTGF*, *EDN1*, *EDNRA*, *ERBB2*, *FGF2*, *FGFR3*, *JAG1*, *PGF*, *PROK2*, *SERPINB5*, *TEK*, *VEGFC* and *VEGFR2*, but up regulated *BAI3* expression, increased cell death, and reduced cell proliferation, led to strong inhibitory effect on angiogenesis (B). Low dose of baicalein also down regulated the transcript levels of *ANG*, *CTGF*, *EDN1*, *EFNA2*, *FGF2*, *FGFR3*, *VEGFC* and *VEGFR2*, but up regulated *MMP9*, reduced cell proliferation, which also resulted inhibition of angiogenesis (B).

Baicalin and baicalein belong to flavonoid family, and they have very similar structures, but their effect on angiogenesis and the mechanism were different. High doses of baicalin and baicalein both had inhibitory effect on angiogenesis in CAM, low dose of baicalein also showed anti-angiogenic activity, but same low dose of baicalin was found to promote angiogenesis (Fig 6). High dose of baicalein down regulated angiogenesis related genes like *ANGPT1*, *CTGF* and growth factors such as *FGF2*, *VEGFC*, increased apoptosis, while also decreasing cell proliferation, ultimately leading to strong inhibitory effect on angiogenesis (Fig 6A). Low dose of baicalein down regulated similar genes as high dose of baicalein did, causing inhibition of angiogenesis through reduction of cell proliferation (Fig 6A). The gene expression profiles in high dose baicalin treated group were complicated, some angiogenesis-related genes such as *ANGPTL4*, *CST3* and *FN1* were up regulated, but other factors including *FGF2* and *VEGFC* were down-regulated, resulting in increased apoptosis, and the inhibition of angiogenesis (Fig 6B). Low dose of baicalin up regulated the expression of several angiogenic genes and growth factors, and led to increased cell proliferation in blood vessels, resulting in pro-angiogenic effect (Fig 6B).

In both cell death and cell proliferation detection, we noticed that the effects of baicalin, baicalein and Huang Qin aqueous extract on cell apoptosis and proliferation were not just observed in epithelium cells of blood vessels, but also in other epithelial and mesenchymal cells in CAM. The results suggested that the cell apoptosis and proliferation induced by baicalin and baicalein may not be vascular-specific. The effects of Huang Qin aqueous extract, baicalin and baicalein on angiogenesis were found to be in a dose-dependent manner, and the inhibitory effect of baicalein was stronger than that of baicalin, these results were consistent with previous studies [41, 56]. The anti-angiogenic effect of Huang Qin extract in a broad dose range may be contributed by the combination of baicalin and baicalein, as well as other components such as wogonin [46, 57]. Compared with inhibitors directly targeting to VEGF or FGF, the chemicals in Huang Qin required relatively high concentrations to achieve their inhibitory effect on angiogenesis [22, 58]. Baicalin showed pro-angiogenic effect at certain doses (10μg/ml-50μg/ml), but the effect is much weaker compared to growth factors like VEGF and FGFs in CAM model [59–61].

Many angiogenic factors and growth factors were down regulated in low or high dose baicalein treated CAM (Figs 5 and 6). *CTGF*, *EDN1*, *FGF2*, *FGFR3*, *VEGFC* and *VEGFR2* were found down regulated in both low and high baicalein treated groups, suggesting that these genes might be baicalein responsive genes. Among these genes, *FGF2* and *VEGF* have been reported [41, 62]. Consistent with our result, Shang et al recently found baicalein suppressed the mRNA expression of *CTGF* in breast cancer cells [63]. Endothelin 1 (EDN1) is a potent vasoconstrictor peptide produced by vascular endothelial cells [64]. Whether the expression of this gene could be affected by baicalin or baicalein has not yet been reported. In our study, we found that *EDN1* expression was reduced in both high and low dose baicalein treated CAM, in addition, *EDN1* and its receptor *EDNRA* were found to be down regulated after high dose of baicalin and baicalein treatments, suggesting *EDN1* is one of the factors responsive to both baicalin and baicalein. Consistent with its pro-angiogenic effect we observed, low dose of baicalin up regulated angiogenic factors like *ANG*, *ANGPT1*, *ANGPTL4*, and growth factors such as *TGFB2* and *VEGFC*, as well as some other angiogenesis related factors (*FN1*, *LITAF*, *MMP9*, *NRP1* and *SERPINF1*). Among all these genes, only *TGF*, *VEGF* and *MMP9* have previously been reported as being influenced by baicalin or baicalein treatment [40, 65, 66].

The gene expression profile in high dose baicalin treated CAM seems to be complicated. Although high dose of baicalin showed strong inhibitory effect on angiogenesis in CAM, and we detected that 6 angiogenesis related genes were up regulated after high dose baicalin treatment, among these up-regulated genes, except for *BAI3*, which is an angiogenesis inhibitor [67], all others (*ANGPTL4*, *CST3*, *FN1*, *MMP9* and *NRP1*) are actually positive regulators of angiogenesis [68–72]. Most of them (*ANGPTL4*, *FN1*, *MMP9* and *NRP1*) were also found to have up regulatory effects on low dose baicalin treated CAM. These results suggested that the increased cell death and inhibitory effect on angiogenesis in high dose baicalin treated CAM might be regulated by different mechanisms other than these angiogenesis related genes.

Although the promoting and inhibitory effects of baicalin on angiogenesis have been reported, no report shows the extract of Huang Qin also has a dual effect [40–44]. In this study, we found that the aqueous extract of Huang Qin had inhibitory effect on angiogenesis in a broad range (8-200mg/ml), but it was also found having a pro-angiogenic effect at low dosage (0.2 mg/ml), which may be mainly due to one of its major components, baicalin (Fig 2D). Baicalin is a glycoside and baicalein is aglycone form of baicalin. Generally, the biological activity of glycosides is mostly due to the aglycone portion, but sometimes the glycoside residue is crucial for their activity [73]. It’s interesting that our results showed only baicalin has dose dependent dual effect on angiogenesis, but the aglycone form baicalein showed dose dependent inhibitory effect alone in CAM model. As to why this happened is unknown, we are able to predict that the epithelial cells in CAM may more efficiently absorb baicalein than baicalin, when low concentration of baicalin was added to the CAM, it went into the cells and regulated angiogenic genes expression, and then perform pro-angiogenic effect. In high concentration of baicalin, certain amount of baicalin might convert into baicalein by glycosidases in eggs. Although we don’t have evidence in CAM model, we do know that both baicalin and baicalein could be detected after oral administration of pure baicalin in rats [74].

In this study, we tested dose dependent effect of herb Huang Qin extract, and its main components baicalin and baicalein on angiogenesis and for the first time our data defined that both Huang Qin aqueous extract and baicalin have dual effect on angiogenesis, and a possible cellular and genetic mechanism was also revealed. In China and some other countries, herb Huang Qin (mostly in formula) is clinically used in patients for both anti-angiogenesis such as inflammation and different kinds of cancer, and pro-angiogenesis such as ischemic diseases treatment [1, 3–5]. Baicalin has recently been used in cosmetics and toothpastes in China [75, 76]. Our data suggest that affecting angiogenesis is one of possible mechanisms of Huang Qin’s function in Traditional Chinese Medicine. According to our results, amount of Huang Qin should be determined based on the disease types, and a new clinical guidance maybe needed. Baicalin concentration in the herb should be carefully monitored for quality control. We strongly suggest that more dose dependent and pharmacokinetics studies in animal models are required before baicalin or baicalein is applied in any clinical trials.

## Supporting Information

S1 Fig Selected CAM photographs showing dose dependent effect of Huang Qin aqueous extract, baicalin and baicalein on angiogenesis. Huang Qin aqueous extract (first column from left), baicalin (middle column) and baicalein (right column) were shown in different columns, the first row was the control for each chemical, all other rows were the photograph images of different concentrations, which was labeled on the left side of the images. The concentrations of baicalin and baicalein were the same in each row, and labeled on the left side of baicalin images. Scale bars in all photographs are 5mm.
(TIF)

S2 Fig Treatment with different dosages of baicalin and baicalein changed CAM morphology. A-F, Selected photographs showing transverse sections of control (A), 40mg/ml Huang Qin aqueous extract (B), 5mg/ml baicalin (C), 5mg/ml baicalein (D), 10μg/ml baicalin (E) and 10μg/ml baicalein (F) treated CAM. G, Comparison of average CAM thickness between different treatments and controls. The scale bars in A-F are 40μm. ce, chorionic epithelium; ae, allantoic epithelium; bv, Blood vessels. Error bars show mean ±SE, and asterisks denote significant differences between each treatment group and control group (*P ≤ 0.05, **P ≤ 0.01). Sample size for each treatment: n = 10.
(TIF)

## References

[1] TPCoPR C. Pharmacopoeia of the People's Republic of China: Beijing Chemical Industry Press; 2005.

[2] Chan FL, Choi HL, Chen ZY, Chan PS, Huang Y. Induction of apoptosis in prostate cancer cell lines by a flavonoid, baicalin. Cancer Lett. 2000;160(2):219–28. 11053652 10.1016/s0304-3835(00)00591-7

[3] Huang WH, Lee AR, Yang CH. Antioxidative and anti-inflammatory activities of polyhydroxyflavonoids of Scutellaria baicalensis GEORGI. Biosci Biotechnol Biochem. 2006;70(10):2371–80. 10.1271/bbb.50698 17031041

[4] Waisundara VY, Hsu A, Huang D, Tan BK. Scutellaria baicalensis enhances the anti-diabetic activity of metformin in streptozotocin-induced diabetic Wistar rats. Am J Chin Med. 2008;36(3):517–40. 10.1142/S0192415X08005953 18543386

[5] Shang X, He X, He X, Li M, Zhang R, Fan P, et al The genus Scutellaria an ethnopharmacological and phytochemical review. J Ethnopharmacol. 2010;128(2):279–313. 10.1016/j.jep.2010.01.006 20064593

[6] Ishimaru K, Nishikawa K, Omoto T, Asai I, Yoshihira K, Shimomura K. Two flavone 2'-glucosides from Scutellaria baicalensis. Phytochemistry. 1995;40(1):279–81. 7546551 10.1016/0031-9422(95)00200-q

[7] Bokhari AA, Syed V. Inhibition of Transforming Growth Factor-beta (TGF-beta) Signaling by Scutellaria baicalensis and Fritillaria cirrhosa Extracts in Endometrial Cancer. J Cell Biochem. 2015;116(8):1797–805. 10.1002/jcb.25138 25683036

[8] Kim EH, Shim B, Kang S, Jeong G, Lee JS, Yu YB, et al Anti-inflammatory effects of Scutellaria baicalensis extract via suppression of immune modulators and MAP kinase signaling molecules. J Ethnopharmacol. 2009;126(2):320–31. 10.1016/j.jep.2009.08.027 19699788

[9] Bochorakova H, Paulova H, Slanina J, Musil P, Taborska E. Main flavonoids in the root of Scutellaria baicalensis cultivated in Europe and their comparative antiradical properties. Phytother Res. 2003;17(6):640–4. 10.1002/ptr.1216 12820232

[10] Yang LX, Liu D, Feng XF, Cui SL, Yang JY, Tang XJ, et al [Determination of flavone for Scutellaria baicalensis from different areas by HPLC]. Zhongguo Zhong Yao Za Zhi. 2002;27(3):166–70. 12774393

[11] Logsdon EA, Finley SD, Popel AS, Mac Gabhann F. A systems biology view of blood vessel growth and remodelling. J Cell Mol Med. 2014;18(8):1491–508. 10.1111/jcmm.12164 24237862 PMC4190897

[12] Lu N, Gao Y, Ling Y, Chen Y, Yang Y, Gu HY, et al Wogonin suppresses tumor growth in vivo and VEGF-induced angiogenesis through inhibiting tyrosine phosphorylation of VEGFR2. Life Sci. 2008;82(17–18):956–63. 10.1016/j.lfs.2008.02.013 18378261

[13] Folkman J. Angiogenesis in cancer, vascular, rheumatoid and other disease. Nat Med. 1995;1(1):27–31. 7584949 10.1038/nm0195-27

[14] Bisht M, Dhasmana DC, Bist SS. Angiogenesis: Future of pharmacological modulation. Indian journal of pharmacology. 2010;42(1):2–8. 10.4103/0253-7613.62395 20606828 PMC2885631

[15] Auerbach R, Lewis R, Shinners B, Kubai L, Akhtar N. Angiogenesis assays: a critical overview. Clinical chemistry. 2003;49(1):32–40. 12507958 10.1373/49.1.32

[16] Tufan AC, Satiroglu-Tufan NL. The chick embryo chorioallantoic membrane as a model system for the study of tumor angiogenesis, invasion and development of anti-angiogenic agents. Current cancer drug targets. 2005;5(4):249–66. 15975046 10.2174/1568009054064624

[17] Griffioen AW, Molema G. Angiogenesis: potentials for pharmacologic intervention in the treatment of cancer, cardiovascular diseases, and chronic inflammation. Pharmacological reviews. 2000;52(2):237–68. 10835101

[18] Hehlgans T, Stoelcker B, Stopfer P, Muller P, Cernaianu G, Guba M, et al Lymphotoxin-beta receptor immune interaction promotes tumor growth by inducing angiogenesis. Cancer Res. 2002;62(14):4034–40. 12124338

[19] Hanahan D, Folkman J. Patterns and emerging mechanisms of the angiogenic switch during tumorigenesis. Cell. 1996;86(3):353–64. 8756718 10.1016/s0092-8674(00)80108-7

[20] Folkman J. Tumor angiogenesis: therapeutic implications. N Engl J Med. 1971;285(21):1182–6. 10.1056/NEJM197111182852108 4938153

[21] Cohen MH, Gootenberg J, Keegan P, Pazdur R. FDA drug approval summary: bevacizumab plus FOLFOX4 as second-line treatment of colorectal cancer. Oncologist. 2007;12(3):356–61. 10.1634/theoncologist.12-3-356 17405901

[22] El-Kenawi AE, El-Remessy AB. Angiogenesis inhibitors in cancer therapy: mechanistic perspective on classification and treatment rationales. Br J Pharmacol. 2013;170(4):712–29. 10.1111/bph.12344 23962094 PMC3799588

[23] Vale PR, Losordo DW, Symes JF, Isner JM. [Growth factors for therapeutic angiogenesis in cardiovascular diseases]. Rev Esp Cardiol. 2001;54(10):1210–24. 11591302 10.1016/s0300-8932(01)76480-9

[24] Deveza L, Choi J, Yang F. Therapeutic angiogenesis for treating cardiovascular diseases. Theranostics. 2012;2(8):801–14. 10.7150/thno.4419 22916079 PMC3425124

[25] Li G, Yu F, Lei T, Gao H, Li P, Sun Y, et al Bone marrow mesenchymal stem cell therapy in ischemic stroke: mechanisms of action and treatment optimization strategies. Neural Regen Res. 2016;11(6):1015–24. 27482235 10.4103/1673-5374.184506 PMC4962565

[26] Losordo DW, Vale PR, Symes JF, Dunnington CH, Esakof DD, Maysky M, et al Gene therapy for myocardial angiogenesis: initial clinical results with direct myocardial injection of phVEGF165 as sole therapy for myocardial ischemia. Circulation. 1998;98(25):2800–4. 9860779 10.1161/01.cir.98.25.2800

[27] Silvestre JS, Levy BI. Angiogenesis therapy in ischemic disease. Arch Mal Coeur Vaiss. 2002;95(3):189–96. 11998334

[28] Wang S, Zheng Z, Weng Y, Yu Y, Zhang D, Fan W, et al Angiogenesis and anti-angiogenesis activity of Chinese medicinal herbal extracts. Life Sci. 2004;74(20):2467–78. 10.1016/j.lfs.2003.03.005 15010258

[29] Sagar SM, Yance D, Wong RK. Natural health products that inhibit angiogenesis: a potential source for investigational new agents to treat cancer-Part 1. Curr Oncol. 2006;13(1):14–26. 17576437 10.3747/co.v13i1.77 PMC1891166

[30] El Sayed KA. Natural products as angiogenesis modulators. Mini Rev Med Chem. 2005;5(11):971–93. 16307528 10.2174/138955705774575291

[31] Sagar SM, Yance D, Wong RK. Natural health products that inhibit angiogenesis: a potential source for investigational new agents to treat cancer-Part 2. Curr Oncol. 2006;13(3):99–107. 17576449 10.3747/co.v13i3.88 PMC1891180

[32] Sato D, Kondo S, Yazawa K, Mukudai Y, Li C, Kamatani T, et al The potential anticancer activity of extracts derived from the roots of on human oral squamous cell carcinoma cells. Mol Clin Oncol. 2013;1(1):105–11. 10.3892/mco.2012.14 24649131 PMC3956248

[33] Yance DR Jr., Sagar SM. Targeting angiogenesis with integrative cancer therapies. Integr Cancer Ther. 2006;5(1):9–29. 10.1177/1534735405285562 16484711

[34] Ye F, Xui L, Yi J, Zhang W, Zhang DY. Anticancer activity of Scutellaria baicalensis and its potential mechanism. J Altern Complement Med. 2002;8(5):567–72. 10.1089/107555302320825075 12470437

[35] Scheck AC, Perry K, Hank NC, Clark WD. Anticancer activity of extracts derived from the mature roots of Scutellaria baicalensis on human malignant brain tumor cells. BMC Complement Altern Med. 2006;6:27 10.1186/1472-6882-6-27 16914050 PMC1560162

[36] Li-Weber M. New therapeutic aspects of flavones: the anticancer properties of Scutellaria and its main active constituents Wogonin, Baicalein and Baicalin. Cancer Treat Rev. 2009;35(1):57–68. 10.1016/j.ctrv.2008.09.005 19004559

[37] Bonham M, Posakony J, Coleman I, Montgomery B, Simon J, Nelson PS. Characterization of chemical constituents in Scutellaria baicalensis with antiandrogenic and growth-inhibitory activities toward prostate carcinoma. Clin Cancer Res. 2005;11(10):3905–14. 10.1158/1078-0432.CCR-04-1974 15897592

[38] Tang W, Sun X, Fang JS, Zhang M, Sucher NJ. Flavonoids from Radix Scutellariae as potential stroke therapeutic agents by targeting the second postsynaptic density 95 (PSD-95)/disc large/zonula occludens-1 (PDZ) domain of PSD-95. Phytomedicine. 2004;11(4):277–84. 10.1078/0944711041495173 15185839

[39] Shao ZH, Li CQ, Vanden Hoek TL, Becker LB, Schumacker PT, Wu JA, et al Extract from Scutellaria baicalensis Georgi attenuates oxidant stress in cardiomyocytes. J Mol Cell Cardiol. 1999;31(10):1885–95. 10.1006/jmcc.1999.1021 10525426

[40] Zhang K, Lu J, Mori T, Smith-Powell L, Synold TW, Chen S, et al Baicalin increases VEGF expression and angiogenesis by activating the ERR{alpha}/PGC-1{alpha} pathway. Cardiovasc Res. 2011;89(2):426–35. 10.1093/cvr/cvq296 20851810 PMC3020130

[41] Liu JJ, Huang TS, Cheng WF, Lu FJ. Baicalein and baicalin are potent inhibitors of angiogenesis: Inhibition of endothelial cell proliferation, migration and differentiation. Int J Cancer. 2003;106(4):559–65. 10.1002/ijc.11267 12845652

[42] Jo H, Jung SH, Yim HB, Lee SJ, Kang KD. The effect of baicalin in a mouse model of retinopathy of prematurity. BMB Rep. 2015;48(5):271–6. 10.5483/BMBRep.2015.48.5.131 25154719 PMC4578566

[43] Miocinovic R, McCabe NP, Keck RW, Jankun J, Hampton JA, Selman SH. In vivo and in vitro effect of baicalein on human prostate cancer cells. Int J Oncol. 2005;26(1):241–6. 15586246

[44] Bassino E, Antoniotti S, Gasparri F, Munaron L. Effects of flavonoid derivatives on human microvascular endothelial cells. Nat Prod Res. 2016:1–4. 10.1080/14786419.2016.1154053 26936689

[45] Huang Y, Zhao K, Hu Y, Zhou Y, Luo X, Li X, et al Wogonoside inhibits angiogenesis in breast cancer via suppressing Wnt/beta-catenin pathway. Mol Carcinog. 2015. 10.1002/mc.22412 26387984

[46] Zhou M, Song X, Huang Y, Wei L, Li Z, You Q, et al Wogonin inhibits H2O2-induced angiogenesis via suppressing PI3K/Akt/NF-kappaB signaling pathway. Vascul Pharmacol. 2014;60(3):110–9. 24534483 10.1016/j.vph.2014.01.010

[47] Reizis A, Hammel I, Ar A. Regional and developmental variations of blood vessel morphometry in the chick embryo chorioallantoic membrane. J Exp Biol. 2005;208(Pt 13):2483–8. 10.1242/jeb.01662 15961734

[48] Gao J, Sanchez-Medina A, Pendry BA, Hughes MJ, Webb GP, Corcoran O. Validation of a HPLC method for flavonoid biomarkers in skullcap (Scutellaria) and its use to illustrate wide variability in the quality of commercial tinctures. J Pharm Pharm Sci. 2008;11(1):77–87. 18445366 10.18433/j39g6v

[49] Rosing H, Man WY, Doyle E, Bult A, Beijnen JH. Bioanalytical liquid chromatographic method validation. A review of current practices and procedures. J Liq Chromatogr R T. 2000;23(3):329–54.

[50] Fogel JL, Thein TZ, Mariani FV. Use of LysoTracker to detect programmed cell death in embryos and differentiating embryonic stem cells. J Vis Exp. 2012;(68). 10.3791/4254 PMC3490301 23092960

[51] Seifert AW, Zheng Z, Ormerod BK, Cohn MJ. Sonic hedgehog controls growth of external genitalia by regulating cell cycle kinetics. Nat Commun. 2010;1:23 10.1038/ncomms1020 20975695 PMC2964453

[52] Ahnfelt-Ronne J, Jorgensen MC, Hald J, Madsen OD, Serup P, Hecksher-Sorensen J. An improved method for three-dimensional reconstruction of protein expression patterns in intact mouse and chicken embryos and organs. J Histochem Cytochem. 2007;55(9):925–30. 10.1369/jhc.7A7226.2007 17478445

[53] Alanentalo T, Asayesh A, Morrison H, Loren CE, Holmberg D, Sharpe J, et al Tomographic molecular imaging and 3D quantification within adult mouse organs. Nat Methods. 2007;4(1):31–3. 10.1038/nmeth985 17143281

[54] Zheng Z, Armfield BA, Cohn MJ. Timing of androgen receptor disruption and estrogen exposure underlies a spectrum of congenital penile anomalies. Proc Natl Acad Sci U S A. 2015;112(52):E7194–203. 10.1073/pnas.1515981112 26598695 PMC4703017

[55] Chomczynski P, Mackey K. Short technical reports. Modification of the TRI reagent procedure for isolation of RNA from polysaccharide- and proteoglycan-rich sources. Biotechniques. 1995;19(6):942–5. 8747660

[56] Chen J, Li Z, Chen AY, Ye X, Luo H, Rankin GO, et al Inhibitory effect of baicalin and baicalein on ovarian cancer cells. Int J Mol Sci. 2013;14(3):6012–25. 10.3390/ijms14036012 23502466 PMC3634505

[57] Song X, Yao J, Wang F, Zhou M, Zhou Y, Wang H, et al Wogonin inhibits tumor angiogenesis via degradation of HIF-1alpha protein. Toxicol Appl Pharmacol. 2013;271(2):144–55. 10.1016/j.taap.2013.04.031 23707765

[58] Cook KM, Figg WD. Angiogenesis inhibitors: current strategies and future prospects. CA Cancer J Clin. 2010;60(4):222–43. 10.3322/caac.20075 20554717 PMC2919227

[59] Cao Y, Linden P, Farnebo J, Cao R, Eriksson A, Kumar V, et al Vascular endothelial growth factor C induces angiogenesis in vivo. Proc Natl Acad Sci U S A. 1998;95(24):14389–94. 9826710 10.1073/pnas.95.24.14389 PMC24383

[60] Bai Y, Leng Y, Yin G, Pu X, Huang Z, Liao X, et al Effects of combinations of BMP-2 with FGF-2 and/or VEGF on HUVECs angiogenesis in vitro and CAM angiogenesis in vivo. Cell Tissue Res. 2014;356(1):109–21. 10.1007/s00441-013-1781-9 24442492

[61] Ribatti D, Maruotti N, Nico B, Longo V, Mangieri D, Vacca A, et al Clodronate inhibits angiogenesis in vitro and in vivo. Oncol Rep. 2008;19(5):1109–12. 18425365 10.3892/or.19.5.1109

[62] Chen SS, Michael A, Butler-Manuel SA. Advances in the treatment of ovarian cancer: a potential role of antiinflammatory phytochemicals. Discov Med. 2012;13(68):7–17. 22284780

[63] Shang D, Li Z, Zhu Z, Chen H, Zhao L, Wang X, et al Baicalein suppresses 17-beta-estradiol-induced migration, adhesion and invasion of breast cancer cells via the G protein-coupled receptor 30 signaling pathway. Oncol Rep. 2015;33(4):2077–85. 10.3892/or.2015.3786 25672442

[64] Yanagisawa M, Kurihara H, Kimura S, Tomobe Y, Kobayashi M, Mitsui Y, et al A novel potent vasoconstrictor peptide produced by vascular endothelial cells. Nature. 1988;332(6163):411–5. 10.1038/332411a0 2451132

[65] Zhang X, Sun CY, Zhang YB, Guo HZ, Feng XX, Peng SZ, et al Kegan Liyan oral liquid ameliorates lipopolysaccharide-induced acute lung injury through inhibition of TLR4-mediated NF-kappaB signaling pathway and MMP-9 expression. J Ethnopharmacol. 2016;186:91–102. 10.1016/j.jep.2016.03.057 27036629

[66] Chung H, Choi HS, Seo EK, Kang DH, Oh ES. Baicalin and baicalein inhibit transforming growth factor-beta1-mediated epithelial-mesenchymal transition in human breast epithelial cells. Biochem Biophys Res Commun. 2015;458(3):707–13. 10.1016/j.bbrc.2015.02.032 25686495

[67] Kee HJ, Ahn KY, Choi KC, Won Song J, Heo T, Jung S, et al Expression of brain-specific angiogenesis inhibitor 3 (BAI3) in normal brain and implications for BAI3 in ischemia-induced brain angiogenesis and malignant glioma. FEBS Lett. 2004;569(1–3):307–16. 10.1016/j.febslet.2004.06.011 15225653

[68] Chong HC, Chan JS, Goh CQ, Gounko NV, Luo B, Wang X, et al Angiopoietin-like 4 stimulates STAT3-mediated iNOS expression and enhances angiogenesis to accelerate wound healing in diabetic mice. Mol Ther. 2014;22(9):1593–604. 10.1038/mt.2014.102 24903577 PMC4435481

[69] Wang B, Sun J, Kitamoto S, Yang M, Grubb A, Chapman HA, et al Cathepsin S controls angiogenesis and tumor growth via matrix-derived angiogenic factors. J Biol Chem. 2006;281(9):6020–9. 10.1074/jbc.M509134200 16365041

[70] Soikkeli J, Podlasz P, Yin M, Nummela P, Jahkola T, Virolainen S, et al Metastatic outgrowth encompasses COL-I, FN1, and POSTN up-regulation and assembly to fibrillar networks regulating cell adhesion, migration, and growth. Am J Pathol. 2010;177(1):387–403. 10.2353/ajpath.2010.090748 20489157 PMC2893681

[71] Padwal M, Siddique I, Wu L, Tang K, Bovin F, Liu L, et al Matrix metalloproteinase 9 is associated with peritoneal membrane solute transport and induces angiogenesis through beta-catenin signaling. Nephrol Dial Transplant. 2016. 10.1093/ndt/gfw076 PMC6251592 27190383

[72] Fantin A, Herzog B, Mahmoud M, Yamaji M, Plein A, Denti L, et al Neuropilin 1 (NRP1) hypomorphism combined with defective VEGF-A binding reveals novel roles for NRP1 in developmental and pathological angiogenesis. Development. 2014;141(3):556–62. 10.1242/dev.103028 24401374 PMC3899814

[73] Kren V, Martinkova L. Glycosides in medicine: "The role of glycosidic residue in biological activity". Curr Med Chem. 2001;8(11):1303–28. 11562268 10.2174/0929867013372193

[74] Liu W, Zhang S, Fu XH, Li HJ, Xiao W, Zhang ZQ. A comparative study of excretion of the components after oral administration of pure baicalin radix scutellariae and scutellariae-paeoniae couple extracts to normal and ulcerative colitis rats. Biomed Res-India. 2015;26(1):13–22.

[75] Gu YX, Yu CH, Zhou ZL. Determination of baicalin in cosmetics and toothpaste by solid-phase extraction and high-performance liquid chromatography. Instrumentation Science & Technology. 2016;44(6):593–602.

[76] Yin HF, Nie HY, Wang S, Zhu WF, Li RM. [Discussing of influence mechanism of Chinese herbal monomer on physical stability of cream]. Zhongguo Zhong Yao Za Zhi. 2014;39(19):3757–63. 25612435

