# Supplementary figures and images for: Dose Dependent Dual Effect of Baicalin and Herb Huang Qin Extract on Angiogenesis
Source: PLoS One. 2016 Nov 30;11(11):e0167125. doi: 10.1371/journal.pone.0167125 (PMC5130244; doi:10.1371/journal.pone.0167125)

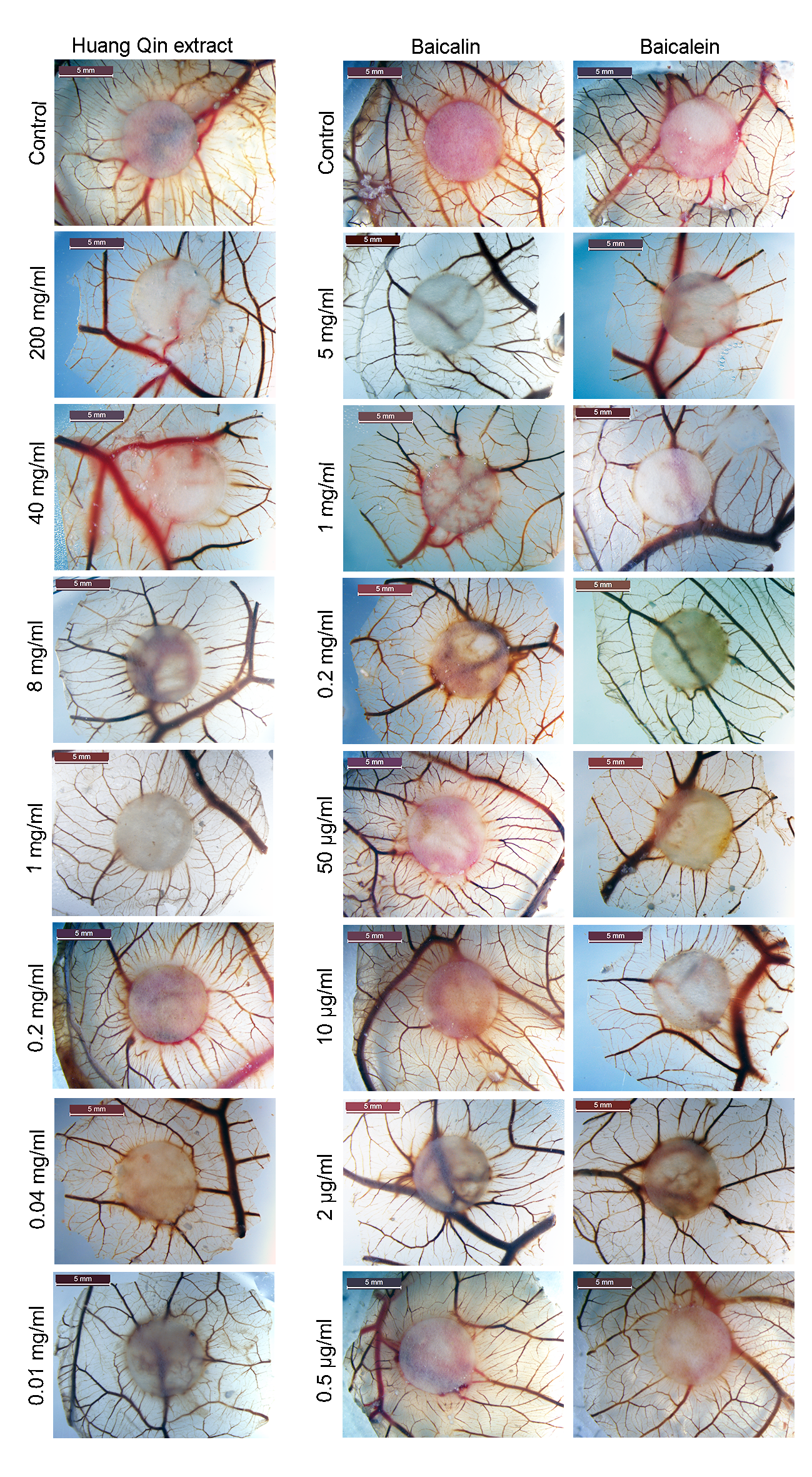

Supplement: S1 Fig — Huang Qin aqueous extract (first column from left), baicalin (middle column) and baicalein (right column) were shown in different columns, the first row was the control for each chemical, all other rows were the photograph images of different concentrations, which was labeled on the left side of the images. The concentrations of baicalin and baicalein were the same in each row, and labeled on the left side of baicalin images. Scale bars in all photographs are 5mm. (TIF) [file pone.0167125.s001.tif]

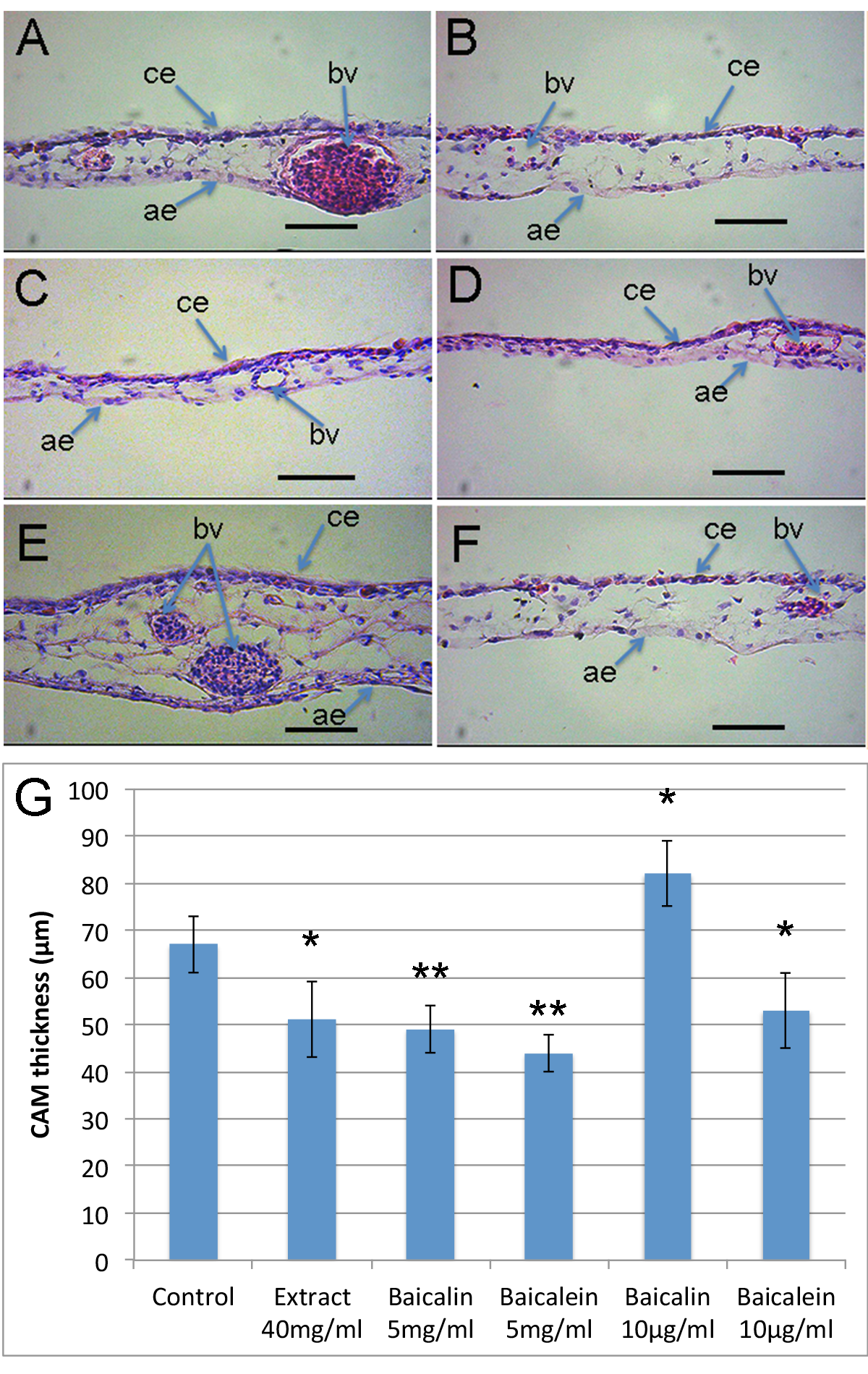

Supplement: S2 Fig — A-F, Selected photographs showing transverse sections of control (A), 40mg/ml Huang Qin aqueous extract (B), 5mg/ml baicalin (C), 5mg/ml baicalein (D), 10μg/ml baicalin (E) and 10μg/ml baicalein (F) treated CAM. G, Comparison of average CAM thickness between different treatments and controls. The scale bars in A-F are 40μm. ce, chorionic epithelium; ae, allantoic epithelium; bv, Blood vessels. Error bars show mean ±SE, and asterisks denote significant differences between each treatment group and control group (*P ≤ 0.05, **P ≤ 0.01). Sample size for each treatment: n = 10. (TIF) [file pone.0167125.s002.tif]
